# Next generation sequencing for clinical diagnostics: Five year experience of an academic laboratory

**DOI:** 10.1016/j.ymgmr.2019.100464

**Published:** 2019-03-01

**Authors:** Paige Hartman, Kenneth Beckman, Kevin Silverstein, Sophia Yohe, Matthew Schomaker, Christine Henzler, Getiria Onsongo, Ham Ching Lam, Sarah Munro, Jerry Daniel, Bradley Billstein, Archana Deshpande, Adam Hauge, Pawel Mroz, Whiwon Lee, Jennifer Holle, Katie Wiens, Kylene Karnuth, Teresa Kemmer, Michaela Leary, Stephen Michel, Laurie Pohlman, Venugopal Thayanithy, Andrew Nelson, Matthew Bower, Bharat Thyagarajan

**Affiliations:** a University of Minnesota Medical School, Duluth, MN, United States of America; b University of Minnesota Genomics Center, University of Minnesota, Minneapolis, MN, United States of America; c Minnesota Supercomputing Institute, University of Minnesota, Minneapolis, MN, United States of America; d Department of Laboratory Medicine and Pathology, University of Minnesota, Minneapolis, MN, United States of America; e Molecular Diagnostics Laboratory, University of Minnesota Health, Minneapolis, MN, United States of America; f Department of Mathematics, Statistics, and Computer Science, Macalaster College, St Paul, MN, United States of America; g Illumina Inc, San Diego, CA, United States of America; h Division of Genetics and Metabolism, University of Minnesota Health, Minneapolis, MN, United States of America; i Invitae, San Francisco, CA, United States of America

**Keywords:** Next generation sequencing, Molecular diagnostics, Panel testing, Diagnostic yield, Variants of uncertain significance, Copy number variation

## Abstract

Clinical laboratories have adopted next generation sequencing (NGS) as a gold standard for the diagnosis of hereditary disorders because of its analytic accuracy, high throughput, and potential for cost-effectiveness. We describe the implementation of a single broad-based NGS sequencing assay to meet the genetic testing needs at the University of Minnesota. A single hybrid capture library preparation was used for each test ordered, data was informatically blinded to clinically-ordered genes, and identified variants were reviewed and classified by genetic counselors and molecular pathologists. We performed 2509 sequencing tests from August 2012 till December 2017. The diagnostic yield has remained steady at 25%, but the number of variants of uncertain significance (VUS) included in a patient report decreased over time with 50% of the patient reports including at least one VUS in 2012 and only 22% of the patient reports reporting a VUS in 2017 (p = .002). Among the various clinical specialties, the diagnostic yield was highest in dermatology (60% diagnostic yield) and ophthalmology (42% diagnostic yield) while the diagnostic yield was lowest in gastrointestinal diseases and pulmonary diseases (10% detection yield in both specialties). Deletion/duplication analysis was also implemented in a subset of panels ordered, with 9% of samples having a diagnostic finding using the deletion/duplication analysis. We have demonstrated the feasibility of this broad-based NGS platform to meet the needs of our academic institution by aggregating a sufficient sample volume from many individually rare tests and providing a flexible ordering for custom, patient-specific panels.

## 1 Introduction

Clinical laboratories have increasingly adopted next generation sequencing (NGS) as a standard for the diagnosis of hereditary disorders. Depending upon the specific focus of the diagnostic laboratory, NGS methods can be used to detect either germline or somatic mutations [2]. Through the use of PCR-based or hybridization-based enrichment strategies, laboratories may focus their clinical analysis on a single gene, multi-gene panels, or all known protein coding genes (exome sequencing) [21]. Since single gene tests and multi-gene panels tend to be the first step in genetic diagnosis of specific clinical diseases. The validity and utility of NGS-based panel testing has been demonstrated for a wide range of conditions including: hearing loss, vision loss, cardiovascular disorders, renal disorders, neurologic disorders, and cancer predispositions [[3], [4], [5],^9^,^16^].

Current short-read based NGS methods have important diagnostic limitations including sequencing of GC-rich sequences, repetitive sequences, and sequences that share high homology with other genes or pseudogenes. Thus, the initial evaluation of a patient suspected of having a hereditary disease may include a combination of both non-NGS based tests and NGS-based gene panels. If this initial targeted testing does not identify a diagnostic finding, whole exome sequencing is often considered [18]. While whole exome sequencing is the most comprehensive, widely available diagnostic option at present, this testing still fails to identify a genetic diagnosis in the majority of patients analyzed [6,19].

While the validity of NGS methods for detection of single nucleotide substitutions and small insertions/deletion mutations is well established, the detection of structural variations and copy number variations (CNVs) is more problematic [1]. Enrichment for targeted panels further may decrease the effectiveness of detection [11]. Hence, a combination of techniques (read depth, read pair, split pair, and assembly based) are used in clinical laboratories for CNV detection [12,17,21]. Since the sensitivity and/or specificity of NGS methods does not yet match other methods (e.g. MLPA and aCGH), some have recommended the concurrent use of multiple methods for CNV detection.

Prior to 2010, the Molecular Diagnostics Laboratory (MDL) at University of Minnesota Health offered a limited menu of 6 genes for Sanger sequencing: *EMD, LMNA, PAH, PAX2, TP53,* and *TYR* that reflected specific expertise at our institution. In 2010, a collaborative effort was undertaken between the MDL, the University of Minnesota Genomics Center (UMGC) and the Minnesota Supercomputing Institute (MSI) to develop a broad-based NGS sequencing menu to serve the diverse sequencing needs of clinicians at our institution. Rather than develop and validate hundreds of individual tests, we developed and validated a single NGS test that captured our entire testing menu [20]. Clinicians could select individual genes, pre-determined panels, or customize a panel from the list of available genes. All samples were processed utilizing identical sequence enrichment methods and a single bioinformatics pipeline. At the final step of the bioinformatics pipeline, the data was restricted to the specific set of genes requested by the ordering physician. This approach allowed our laboratory to offer a comprehensive sequencing menu utilizing a single wet-bench workflow and bioinformatics pipeline [13,20]. Subsequently, a custom coverage-based CNV detection method was developed and validated to complement the detection of sequence variants [12].

This is a retrospective review of 2509 NGS-based clinical genetic tests performed at our laboratory from 2012 to 2017 to illustrate the implementation of NGS based variant detection and CNV analysis on a broad scale at an academic clinical laboratory. The types of gene panels ordered, distribution of orders and detection rates by specialty and specific phenotypes and CNV results are discussed.

## 2 Methods

### 2.1 Study population

This study is a retrospective review of 2509 sequencing tests performed at University of Minnesota Health Molecular Diagnostics Laboratory (MDL) from August 2012 to December 2017. A de-identified data set was analyzed to extract relevant data. Only patients referred to our laboratory for appropriate diagnostic testing following a clinical examination were included in this study. Patients referred to our laboratory for identification of mutation carrier status or predictive testing were excluded from this analysis. This study was reviewed and deemed exempt by the Institutional Review Board of the University of Minnesota. A detailed description of the specific genes offered is provided in Supplementary Table 1 and gene panels offered within each clinical specialty is provided in Supplementary Table 2.

### 2.2 Overview of processing

From August 2012 to March 2014, 349 patient samples were processed using a custom SureSelect sequence capture panel (Agilent Technologies Inc., Santa Clara, CA) targeting the coding region of 568 clinically relevant genes [20]. Prepared libraries were sequenced on an HiSeq 2000 instrument (Illumina Inc., San Diego, CA). Subsequently from April 2014 to September 2017, 2058 patient samples were processed using the TruSightOne 4813 gene capture kit (Illumina Inc., San Diego, CA) and sequenced on an HiSeq 2500 instrument in rapid mode (Illumina Inc., San Diego, CA) [10]. Finally, 102 samples processed from October 2017 to December 2017 were processed using a combination of the TruSightOne Expanded sequence capture (6735 genes) (Illumina Inc. San Diego, CA) and a custom designed capture targeting an additional 205 clinically relevant genes and 429 clinically relevant non-coding regions (deep intron mutations, untranslated regions, promotors, non-coding RNA). These samples were sequenced on an Illumina HiSeq 2500 (Illumina Inc., San Diego, CA) in high output mode.

Following sequencing, alignment and variant detection were performed in a custom cloud-based informatics pipeline as described previously [13]. De-identified sequencing data is uploaded to a cloud-based supercomputing instance and mapped using a BWA alignment tool and genotyped using a GATK pipeline. At the final step of this informatics pipeline, the coverage metrics and VCF file are restricted to output only data specific to the genes ordered by the clinician.

Though the average coverage of clinically ordered gene panels were typically in the range of 200×, there are some genomic regions where coverage dropped below our minimum threshold to interpret NGS data (defined initially as <20× coverage and later revised to <15× coverage). These low coverage regions were not analyzed by NGS but were sequenced, when possible, by Sanger sequencing using custom designed primers specific to low coverage regions. Any regions that did not meet minimum NGS coverage and could not be analyzed by Sanger sequencing were specifically identified in patient reports. Though initially we confirmed all clinically reported pathogenic/likely pathogenic variants or variants of possible clinical significance using Sanger sequencing, more recently we only confirm a subset of variants that do not meet pre-determined quality criteria [10] We also developed custom Sanger sequencing for targeted follow up testing of family members.

A custom coverage-based copy number variation (CNV) algorithm was developed beginning in 2013 and CNV-Random Forest (CNV-RF), was more broadly implemented in 2015 [12]. Briefly, CNV-RF is a read depth based method that was optimized for detection of deletions and duplications using targeted NGS data. This method compares the coverage for a particular genomic region in the sample as compared to a control to identify deletions and duplications. This method was clinically validated to detect deletions as small as 180 bp and duplications as small as 300 bp. This implementation allowed for concurrent detection of clinically significant CNVs from the same data sets used for genotyping.

### 2.3 Panel development

Clinical panels were developed through a collaborative process involving both MDL and clinical experts at our institution. Panels were initially developed based upon a review of medical literature and public databases (www.omim.org). These panels were customized with input from clinical collaborators to reflect the specific ordering practices at our institution. The content of the panels was continuously updated both as new disease genes were identified and as we implemented larger sequence capture platforms. Thus, the content of an individual panel varied significantly over the time course of this retrospective study. Based upon clinical evaluation of the patient, ordering providers had the flexibility to choose from these pre-defined panels, to customize panels with additional genes, or to order multiple panels for a single patient.

### 2.4 Variant interpretation

Variant interpretation was initially performed through an unstructured process involving review by both genetic counselors and molecular pathologists. Discrepancies in interpretation were resolved through discussion and consensus conferences. With the publication of formal interpretation guidelines [15], a structured variant interpretation process was put in place in 2016. A published online tool was utilized to facilitate variant classification [7].

In addition to the 5 variant categories described in the guidelines, the laboratory employed an additional category termed “variants with possible clinical significance” to describe variants strongly suspected to be pathogenic but lacking sufficient evidence to formally be categorized as pathogenic or likely pathogenic by ACMG/AMP criteria. In many cases, segregation analysis in the family could provide the additional evidence needed to re-classify these variants as pathogenic, likely pathogenic, or likely benign. The variants were highlighted on clinical reports along with recommendations for additional evaluations that could help to clarify the clinical significance of these variants.

### 2.5 Data analysis

A de-identified data set was reviewed, and the following information was collected to determine aggregate statistics: test requested, number of genes requested, diagnostic findings, number of variants of uncertain significance reported in the clinical report, and distribution of orders by clinical specialty. Tests were considered diagnostic when a single pathogenic or likely pathogenic mutation was identified in a gene with a known autosomal dominant or X-linked pattern of inheritance, or when two pathogenic or likely pathogenic variants were identified in a gene with a known recessive pattern of inheritance. Cases were considered to have “possible diagnostic findings” either when variant(s) with possible clinical significance were identified or when a single pathogenic/likely pathogenic variant was identified in an autosomal recessive disease gene. A negative finding was defined as a case with either no reported variants or only variants of uncertain significance (VUS).

## 3 Results

### 3.1 Diagnostic yield

**Fig. 1:**
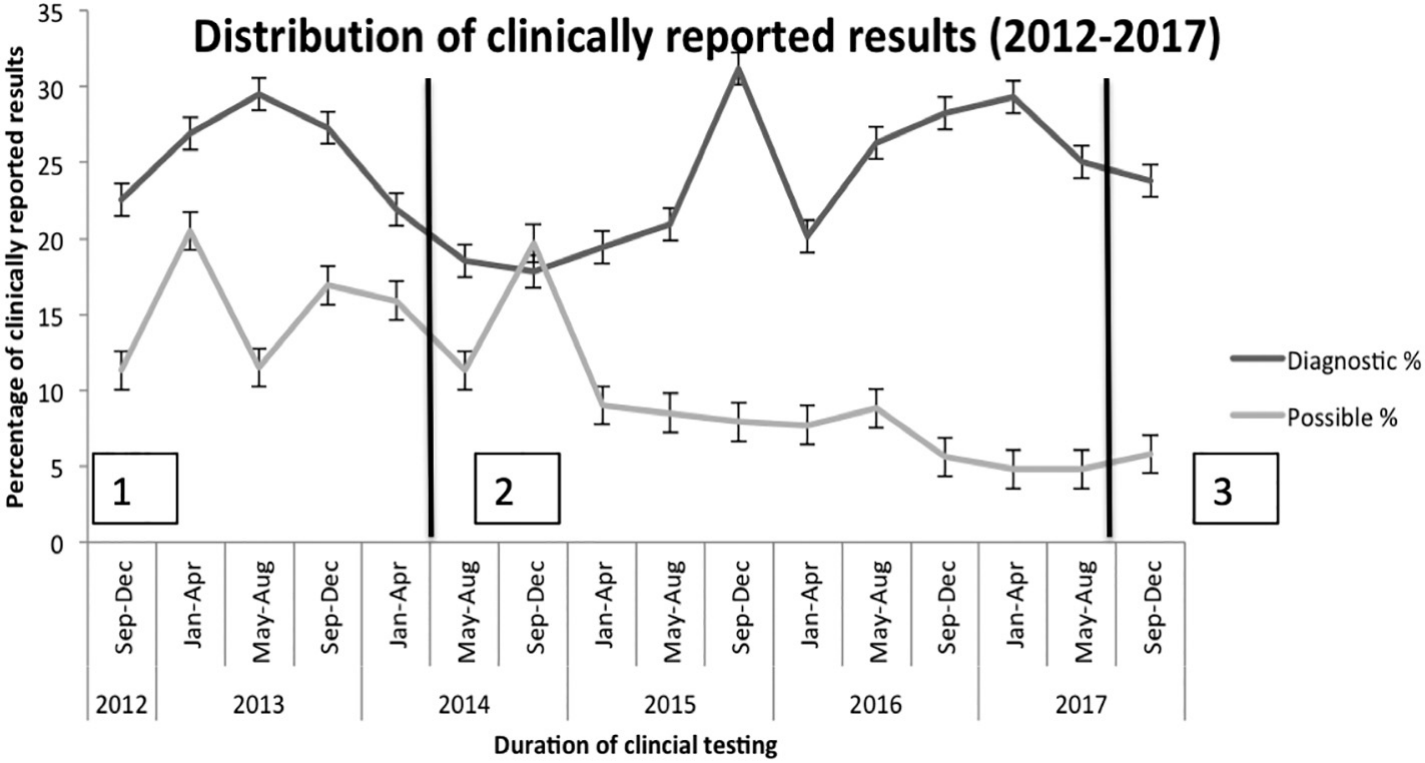
Longitudinal trend in diagnostic yield in targeted NGS panels among 2509 clinical samples (2012–2017): The proportion of samples reported with diagnostic findings and as negative remained relatively stable over time while there was a decrease in the number of samples reported with possible diagnostic findings from 16.3% in 2013 to 5.13% in 2017. 1 = SureSelect NGS panel (Agilent Inc.) 2 = Trusight One targeted NGS panel (Illumina Inc.) 3 = TruSight One NGS panel (Illumina Inc.)

Throughout the first six years of testing, 2509 specimens were assayed for mutation detection, deletion/duplication detection or a combination of both. Annual sample volume increased steadily over the first three years from 71 samples in 2012, 233 samples in 2013, 454 samples in 2014 to 604 samples in 2015. The sample volume was relatively stable since 2015. Overall, a diagnostic finding was identified in 24.10% (24.08%–24.14%) of samples, a possible diagnostic finding was identified in 9.56% (9.54%–9.58%) of samples, and 66.33% (66.31%–66.36%) of samples were reported as negative. The proportion of samples reported with diagnostic findings and as negative remained relatively stable over time (Fig. 1) while there was a decrease in the number of samples reported with possible diagnostic findings from 16.3% in 2013 to 5.13% in 2017. This decrease in number of samples with possible diagnostic findings was not related to the various versions of the clinical assay performed over the time period (Fig. 1).

### 3.2 Variants of uncertain significance

**Fig. 2:**
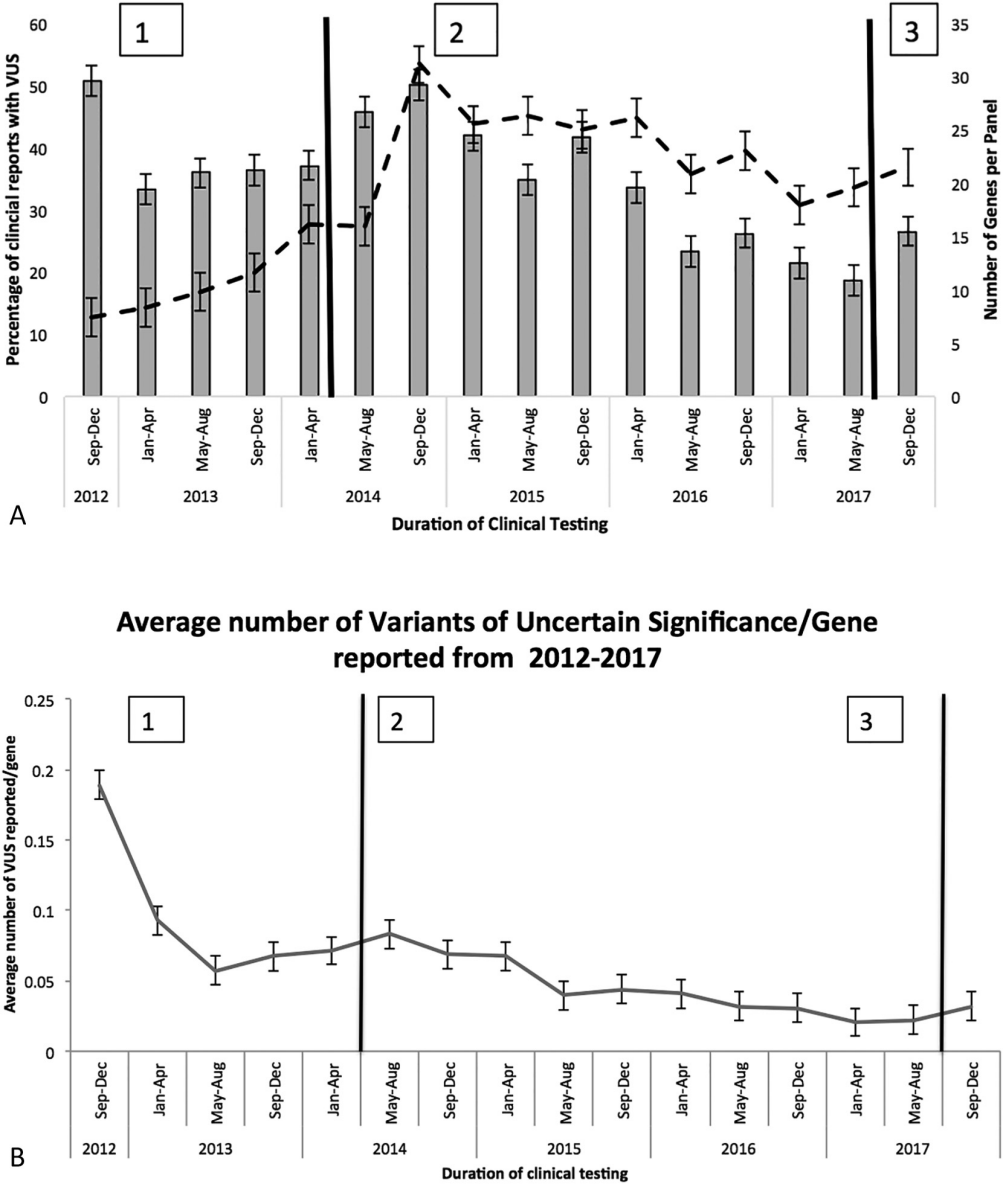
A: Longitudinal trend in the percentage of clinical NGS reports with VUS and the number of genes tested (2012–2017): The proportion of reports with variants of uncertain significance (VUS) decreased from 50.70% in 2012 to 22.12% in 2017 (solid bars) while the average number of genes analyzed per report increased during the same time period from 8 genes/panel in 2012 to 25 genes/panel in 2017 (dotted line), with the largest increase in genes/panel happening in 2014 due to the change from the SureSelect capture panel (Agilent Inc.) to the TruSight One panel (Illumina Inc.). B: Number of VUS reported per gene analyzed, the number of VUS identified per gene analyzed decreased 0.19 VUS reported per gene analyzed in 2012 to 0.03 VUS reported per gene analyzed in 2017. 1 = SureSelect NGS panel (Agilent Inc.) 2 = Trusight One targeted NGS panel (Illumina Inc.) 3 = TruSight One NGS panel (Illumina Inc.)

The proportion of reports with variants of uncertain significance (VUS) has decreased over time (50.70% in 2012 and 22.12% in 2017; p = .002) despite average number of genes analyzed per report increasing over time (Fig. 2A) (8 genes/panel in 2012 vs. 25 genes/panel in 2017), with the largest increase in genes/panel happening in 2014 when we changed from the SureSelect capture panel (Agilent Technologies Inc., Santa Clara, CA) to the TruSight One panel (Illumina Inc., San Diego, CA). When accounting for the number of genes analyzed, the number of VUS identified per gene analyzed has decreased over time (Fig. 2B) with an average of 0.19 VUS reported per gene analyzed in 2012 to 0.03 VUS reported per gene analyzed in 2017. This translates into one reported VUS for approximately every 5 genes analyzed in 2012 compared to one VUS for approximately every 33 genes analyzed in 2017; (p < .001) (Fig. 2B).

### 3.3 Copy number variation

Since broad roll out of the deletion/duplication assay in 2015, the proportion of samples analyzed for CNV increased rapidly from 10% to 72% between 2015 and 2017, and the rate has remained relatively stable since that time. Thus, a majority of all genes panels ordered in our laboratory currently include CNV analysis in addition to standard variant analysis. CNV testing was requested in a total of 728 samples (29%) with pathogenic CNVs detected in 65/728 (9%) of cases. These CNVs included whole gene duplications (n = 22), heterozygous whole gene or partial gene deletions (n = 35), homozygous whole gene deletions (n = 7), and complex rearrangement characterized by partial duplication and partial deletion of a single gene (n = 1). CNVs in 5 genes (*PMP22*, *FANCA*, *HNF1B*, *STRC*, *NPHP*1) accounted for 68% of the detected copy number alterations (44/65). The remaining 21 CNVs were distributed across 16 different genes: *ADGRV1, COL7A1, CLN3, CTNS, CYBB, CYP7B1, FBN1, FBP1, FLNA, LMNB1, NR3C2, OTC, PAH, PAH, SPG4, TGFB2, TGFB2,* and *USH1C.*

### 3.4 Distribution of orders by clinical specialty

**Fig. 3:**
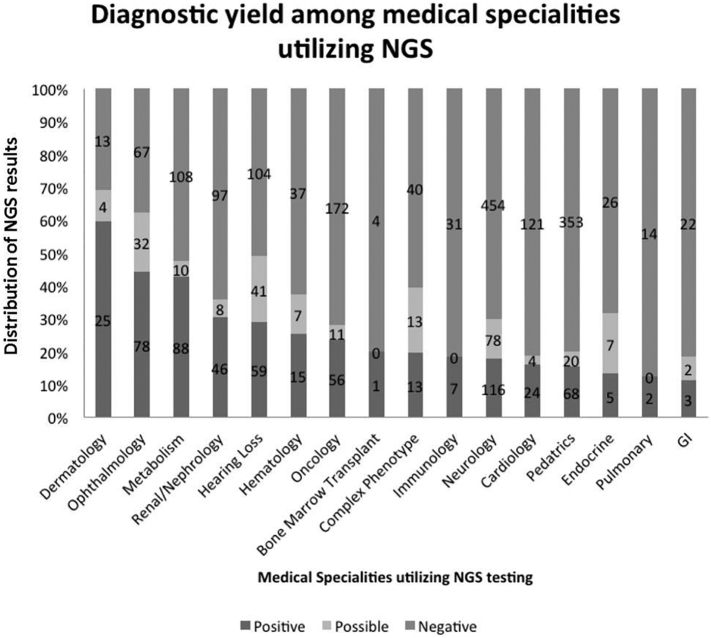
Utilization and diagnostic yield of targeted NGS panels across clinical specialties: The most common specialties requesting targeted NGS testing were neurology (n = 676) and pediatrics (n = 587), accounting for 50% of all targeted NGS panels ordered at the University of Minnesota. The diagnostic yield was highest in dermatology (60%), hearing loss (49%) and ophthalmology (42%) while the diagnostic yield was lowest in gastrointestinal diseases and pulmonary diseases (10% detection yield in both specialties). The numbers within each vertical bar represent the total number of samples in each category (positive, possible or negative) within each medical specialty. Three samples were excluded as they could not be readily classified into one of the defined clinical areas.

The distribution of orders by clinical specialty is presented in Fig. 3. The most common specialties requesting testing were neurology (n = 676) and pediatrics (n = 587), collectively accounting for half of all orders. The next most common specialties included oncology (n = 242), hearing loss (n = 221), metabolism (n = 217), cardiology (n = 194), and ophthalmology (n = 190) (Fig. 3).

### 3.5 Positivity by panel

Table 1 provides a summary of the diagnostic yield for commonly ordered gene panels (i.e.) gene panels that were ordered ≥10 times from 2012 to 2017. Roughly one third of all tests (n = 843) ordered in our laboratory were infrequently ordered and diagnostic yield for these panels was not calculated. As expected, single gene tests for highly specific conditions and small gene panels with discretely described phenotypes had higher rates of diagnostic findings. For example, *PAH* sequencing for phenylketonuria had a consistently high diagnostic rate of 96% in 52 samples, sequencing of the *COL4A3, COL4A4*, and *COL4A5* genes in Alport syndrome had a diagnostic yield of 48% in 48 samples and the commonly ordered comprehensive hearing loss panel, most commonly ordered as a panel with 2–149 genes had a diagnostic rate of 50% in 173 samples (Table 1). In contrast, the diagnostic yield for the Ehlers Danlos syndrome panel, most commonly ordered as a panel with 13–16 genes, was very low at 6% (Table 1).

**Table 1:** Diagnostic yield for commonly ordered gene panels (n ≥ 10 samples) at the University of Minnesota from 2012 to 2017.

Panel	Total cases	Number of genes^a^	Diagnostic findings	Possible diagnostic findings	Negative findings	Diagnostic yield (%) (Diagnostic + Possible diagnostic)
Phenylketonuria	52	1	49	1	2	96
Fanconi anemia	39	1–18	30	4	5	87
Epidermolysis bullosa	27	1–13	20	3	4	85
Retinal dystrophy panel	69	32–315	40	15	14	80
Adrenoleukodystrophy	18	1	13	1	4	78
Albinism	42	1–24	20	12	10	76
Congenital hyperinsulinism	12	7–14	3	5	4	67
Craniosynostosis	13	1–20	5	2	6	54
Hearing loss (all subpanels combined)	173	2–149	50	37	86	50
Achondroplasia	10	1	5	0	5	50
Congenital myopathy	35	1–29	9	8	18	49
Alport syndrome	48	3	20	3	25	48
Stickler Syndrome	13	1–6	5	1	7	46
Ataxia/Hereditary Spastic Parapresis	23	28–101	3	7	13	43
Hereditary spastic paraparesis	26	1–74	7	4	15	42
Limb girdle muscular dystrophy	24	1–36	6	4	14	42
Ataxia	25	21–67	3	7	15	40
Carnitine acetyltransferase deficiency	10	1	1	3	6	40
Cystic fibrosis	10	1	3	1	6	40
Noonan syndrome	40	5–22	15	0	25	38
Hereditary hemorrhagic telangiectasia	11	1–4	4	0	7	36
Periodic paralysis syndromes	11	2–5	3	1	7	36
Polycystic kidney disease	11	1–9	3	1	7	36
Charcot Marie Tooth	117	1–58	32	9	76	35
Glycogen storage disease	17	1–25	5	0	12	29
Complex neurologic	45	6–266	4	8	33	27
Osteogenesis imperfecta	34	2–16	7	2	25	26
Hypophosphotasia	35	1	9	0	26	26
Malignant hyperthermia	12	1–2	2	1	9	25
Vascular malformations	12	1–5	2	1	9	25
X-linked Intellectual disability	16	43	3	1	12	25
Disorders of sex development	22	16–63	3	2	17	23
Myofibrillar myopathy	14	5–13	0	3	11	21
Parkinson's disease	27	2–25	3	2	22	19
Cornelia de Lange syndrome	11	5–8	2	0	9	18
Aortopathy (TAAD)	60	2–27	6	4	50	17
Periodic fever panel	18	7–9	3	0	15	17
Urea Cycle	12	1–6	2	0	10	17
Dyskeratosis congenita	13	7–24	2	0	11	15
Focal segmental glomerulosclerosis	20	8–40	2	1	17	15
Marfan syndrome	38	1–3	2	3	33	13
Li Fraumeni syndrome	25	1–3	3	0	22	12
Macrocephaly/Overgrowth	10	3–18	1	0	9	10
Hereditary breast/ovarian cancer	126	2–18	11	1	114	10
Connective tissue disorder	48	2–29	2	2	44	8
Developmental eye panel	12	14–31	1	0	11	8
Renal coloboma syndrome	13	1	1	0	12	8
Ehlers Danlos syndrome	111	1–16	5	2	104	6
Dystonia	17	1–18	1	0	16	6
Motor neuron disease	19	5–85	1	0	18	5
Myoclonus dystonia	20	1–3	1	0	19	5

a The number of genes requested for each panel varied both due to the addition of genes to panels over time and due to customization by ordering providers based upon the specific patients clinical presentation and/or family history.

### 3.6 Sanger sequencing

Sanger sequencing was initially used from 2012 to 2015 to confirm all pathogenic or likely pathogenic variants in clinical reports (n = 294); however after 2015 the laboratory implemented a policy to only confirm variants by Sanger sequencing if they do not not meeta set of five quality thresholds [10]. This policy allowed for 81% (237/294) of diagnostic variants to be reported without Sanger confirmation. Subsequently, Sanger sequencing of variants has only been used to confirm the presence of mutations not meeting these criteria in probands and to provide targeted mutation confirmation or exclusion in family members.

Sanger sequencing was also used to supplement NGS data in low coverage regions. Initially in 2012, any coding base that did not achieve 20× coverage was analyzed by Sanger sequencing. Two modifications were made to these criteria after 2014: (a) Due to the increase in panel sizes with the implementation of the larger TrusightOne capture, Sanger sequencing for low coverage regions was not performed in large panels (>25 genes) and (b) for panels with ≤25 genes, Sanger sequencing was only performed for regions that did not achieve a minimum of 15× coverage. Regions covered at between 15-20× were specifically noted on clinical reports as regions with reduced sensitivity for mutation detection. Across the 2509 samples reviewed, Sanger sequencing was performed for 6584 low coverage regions. This supplementary sequencing yielded two pathogenic mutations that were not detected by NGS (one each in *CFTR* and *GPR143*). In both cases, the variants occurred in regions with zero coverage by the NGS assay.

## 4 Discussion

We demonstrate the feasibility of implementing a flexible, broad-based NGS based sequencing panel to meet the clinical genetic testing needs at the University of Minnesota. We employed several strategies to ensure successful implementation of this approach. First, we worked closely with the clinicians in various clinical specialties to ensure that genes and panels relevant to their clinical practice are included in the NGS panel. Second, in addition to building readily orderable single gene tests and pre-defined disease-specific panels, we also provided the option to order custom gene panels. Custom panel ordering allows our clinicians to order test panels based on the specific phenotype of their patients, often combining standard panels, and adding or removing specific genes from panels as necessary. Lastly, we developed an ongoing mechanism to curate and maintain list of newly identified clinically relevant genes that we include in regularly scheduled updates to our gene panels.

As evidenced by both the rapid increase in sample volume after the initial implementation and the sustained high volume of samples processed in our laboratory, these processes have generated both initial enthusiasm and have sustained interest from clinicians in using the NGS panel offered at our institution. The procedures implemented in our laboratory have allowed more of the genetic testing to be performed within our institution and resulting in a 695% increase in the numbers of genetic tests ordered for inherited diseases in our laboratory and resulted in a corresponding decrease in the volume of genetic testing being sent to commercial reference laboratories.

While our policy of providing flexibility to the ordering clinicians to choose appropriate gene sets based on the patients clinical presentation and providing a limited number of pre-made gene panels has been very successful in encouraging clinicians to use genetic services provided by our laboratory, this policy of allowing ordering of flexible targeted gene panels limits the potential for identifying mutations in recently identified new disease genes and/or reduce diagnostic yield if only partial gene panels are ordered. To overcome this limitation, we offer analysis of other genes (included in our large panel but not clinically ordered) as a supplemental test for a nominal cost that the clinicians can order if they consider additional testing to be clinically relevant.

Our overall approach has been remarkably adaptable over time. Since the initial clinical validation, we have adapted this testing platform across three different sequencing instruments and enrichment methods, and have implemented a custom CNV detection algorithm. There are several advantages to implementing a single broad based gene panel as compared to implementing focused discrete disease specific panels both during initial introduction of the clinical test and ongoing maintenance of the clinical test. First, the cost and time investment for clinical validation is similar irrespective of the size of the gene panels. Hence, designing and validating a single large clinical assay is very cost effective as compared to designing and validating multiple small disease specific panels. Second, given the rapid pace at which new disease genes are being diagnosed, having a single clinical test allows rapid upgrading of several disease panels simultaneously. Our clinical assay is upgraded approximately every 12–18 months to ensure the clinical test includes all clinically relevant genes across multiple clinical disciplines. Lastly, employing a broad based approach dramatically increases the number of samples available for sequencing and allows us to use large scale sequencers (e.g. HiSeq or NovaSeq) that dramatically reduce sequencing costs as compared to smaller desktop sequencers. For example though our annual sample volume averages around 600 samples, a majority of the individual disease specific panels have an annual volume of >50 samples. Thus, having large sample volumes by meeting the sequencing needs of diverse clinical specialties has helped drive down sequencing costs and allowed us to remain on the forefront of rapid translation of new research findings into clinical practice.

Despite significant increases in the number of available genes over time (568 genes to 6940 genes), the diagnostic yield has remained steady at 24.1%. These data support the notion that the majority of the diagnostic yield comes from a small core groups of genes for many phenotypes. While adding newly discovered genes may facilitate the diagnosis in specific patients and allow for expansion of the test menu to include newly described phenotypes, it did not have a significant impact on the overall diagnostic yield of many targeted phenotype panels. Our overall observed diagnostic yield of 24% is also similar to the published diagnostic yield from large exome sequencing studies [18] further supporting the notion that increasing the number of genes sequenced does not always result in additional diagnostic information. However, the overall diagnostic yield across all clinical areas is not an accurate metric regarding the utility of sequencing as there is a wide range of diagnostic yields within different specialties (Fig. 3). While dermatology, hearing loss and congenital retinal disorders have a high diagnostic yield (42%–60%), other specialties such as pulmonary, cardiology and gastrointestinal systems have low diagnostic yield (<30%). Since increasing the size of the disease panel has not greatly improved diagnostic yields, evaluating structural genetic variation and/or regulatory gene variation may be needed to improve diagnostic yields in certain clinical specialties.

In contrast to the relatively stable diagnostic yield, we observed a decrease in VUS over time. The initial steep decline (2012−2013) was due to early adjustments in the types of variants that were deemed necessary to include in patient reports. Subsequently, the proportion of reports with a VUS continued to decrease over time, despite a significant increase in the number of genes ordered per test. This decrease in reported VUS is in line with the experience across many clinical laboratories and is predominantly due to the rapid increase in the large amount of genetic information available in the public domain. Whole exome and whole genome data from hundreds of thousands of phenotypically normal individuals in large public databases such as ExAC and gnomAD [8] together with large number of variants reported from clinical laboratories deposited in freely available databases such as ClinVar allows for more definitive classification of rare variants. With the anticipated release of whole genome information for additional hundreds of thousands of individuals in the near future (e.g. TopMED database), we anticipate this trend of decreasing numbers of VUS reported will continue. In addition, developing a structured variant interpretation protocol based upon published guidelines from ACMGG and AMP [14,15] allowed us to standardize our variant interpretation pipelines and also likely contributed to the ability to better classify variants in pathogenic/likely pathogenic or benign/likely benign categories.

While the initial goal of this effort was to replace Sanger sequencing in our laboratory as a primary method for diagnosis, the overall use of Sanger sequencing actually increased dramatically with the implementation of NGS due to targeted testing of family members, confirming selected variants by Sanger sequencing prior to reporting them and using Sanger sequencing for regions of low NGS coverage.

These data represent the experience at a single institution and thus reflect the specific patient populations referred to our institution and the practice and ordering behaviors of specific clinicians and departments. While the conclusions about the diagnostic yield of specific panels may not be generalizable across other institutions and patient populations, we describe a cost effective approach for implementation of NGS based sequencing that can be adapted to meet the sequencing needs across diverse institutions. Initial clinical implementation of NGS testing was possible due the strong interest in utilizing NGS based testing from neurologists at our institution and formed the foundation for obtaining initial funding from the Institute for Translational Neuroscience (ITN) to develop clinical NGS infrastructure that could support future translational research. We utilized these funds to develop a broad based NGS assay that fully met the needs of the neurologists and ITN but also allowed us to expand our test menu beyond one clinical specialty. The ability to offer a NGS based test to a broad range of specialties was a critical step in convincing hospital administrators to invest in further development of clinical NGS infrastructure. Since a large number of send-out genetic tests could now be performed in-house hospital administrators could see tangible benefits from both reduction in costs of send-out testing and additional revenue from the genetic testing that was being performed in-house. In addition, this has provided our academic institution to provide a unique and local genetic testing service not otherwise available in our market.

We have demonstrated the feasibility of implementing a broad-based cost effective NGS based assay to meet the diverse sequencing needs at the University of Minnesota. Over the past 5 years, this NGS platform has provided a flexible test menu that allows clinicians to selectively choose the genes they want tested, while retaining a consistent pipeline for multiple rare genetic tests. While this “one-size-fits-all” approach has allowed us to meet the needs for a significant proportion of genetic testing at our institution, detection of variants in regions with significant homology or have pseudogenes (e.g.) *SMN1*, *PMS2* and *CYP21A2* or regions with recurrent structural variation/repeat expansions cannot be detected using the methods described here and will require specialized approaches. Overall, this platform has allowed us to produce a sufficient volume of rare tests while remaining cost effective for our academic institution.

The following are the supplementary data related to this article.

Supplementary Table 1 Clinically available genes and associated conditions. Where possible, condition names are consistent with nomenclature used in the Online Mendelian Inheritance in Man (omim.org) database. Genes with more than one clinical association are listed multiple times in the table. Supplementary Table 1

Supplementary Table 2 Available panels. Panel names are provided along with a comma delimited list of genes included in the panel. The genes within the panel, and the associated conditions from Supplementary Table 1 are also provided for each panel. Supplementary Table 2

## Funding sources

This work was supported in part by the Bob Allison Ataxia Research Center and the Institute for Translational Neuroscience at the University of Minnesota.

## Disclosures

None of the authors have any significant conflicts of interest or disclosures to report.
